# Self-reported pediatricians' management of the well-appearing young child with fever without a source: first survey in an European country in the anti-pneumococcal vaccine era

**DOI:** 10.1186/1471-2458-9-300

**Published:** 2009-08-19

**Authors:** Elena Chiappini, Luisa Galli, Francesca Bonsignori, Elisabetta Venturini, Nicola Principi, Maurizio de Martino

**Affiliations:** 1 Department of Pediatrics, University of Florence, Florence, Italy; 2 Department of Maternal and Pediatric Sciences, University of Milan, Fondazione IRCCS Ospedale Maggiore Policlinico, Mangiagalli e Regina Elena, Milan, Italy

## Abstract

**Background:**

Recent studies suggest a substantially reduced risk of invasive bacterial infection in children vaccinated with heptavalent pneumococcal conjugate vaccine (PCV). To investigate whether the introduction of PCV might affect clinical decision making, we conducted a cross-sectional survey aimed at Italian Pediatric physicians.

**Results:**

The study included 348 (46.5%) primary care pediatricians; 251 (36.4%) hospital pediatricians, and 139 (20.1%) pediatric residents. In an hypothetical scenario, a well-appearing 12-month-old child with fever without source would be sent home with no therapy by 60.7% (419/690) of physicians if the child was not vaccinated with PCV. The proportion increased to 74.2% (512/690) if the child had received PCV (P < 0.0001). Also, physicians would obtain blood tests less frequently in the vaccinated than in unvaccinated children (139/690 [20.1%] *vs*. 205/690 [29.7%]; P < 0.0001), and started empiric antibiotic therapy less frequently (3.0% *vs*. 7.5%; P < 0.0001). In the hypothetical event that white blood cell count was 17,500/μL, a significantly lower proportion of physicians would ask for erythrocyte sedimentation rate (P < 0.017), C reactive protein (P < 0.0001), blood culture (P = 0.022), and urine analysis or dipstick (P = 0.028), if the child had received PCV. Only one third of participants routinely recommended PCV.

**Conclusion:**

Our data suggest that implementation of educational programs regarding the proper management of the febrile child is needed.

## Background

The management of children with a fever without a source remains controversial [1], and it is becoming even more so after the introduction of the hepta-valent pneumococcal conjugate vaccine (PCV) [1]. It has been calculated that the widespread use of PCV is associated with a decreased risk of bacterial infection from about 2–5% to less than 1% in well-appearing young children with a fever without a source [1]. Given this minimal risk, some authors hope that the management of these children will become a non-entity in the near future, and screening blood tests (e.g. complete blood cell count with differential, blood culture), and empiric antibiotic treatment will become unnecessary [2].

However, available data are insufficient to support modifications of current clinical practice guidelines. Also of concern, increasing rates of infections due to antimicrobial resistant pneumococcal serotypes not-included into the heptavalent PCV have been reported [3-6]

To the best of our knowledge two surveys previously investigated physicians' self-reported attitudes toward the management of young children presenting with a fever without a source, since the introduction of PCV [7,8]. Both the studies were conducted in the United States and found that pediatricians would order fewer complete blood cell counts, blood cultures, and urine tests, and would prescribe less antibiotics in the event that the child is vaccinated with PCV [7,8]. Thus the introduction of PCV seems to affect pediatricians' decision making in the United States. No similar study has been conducted in an European country. Therefore, we carried out a cross-sectional survey on a sample of Italian pediatricians regarding the management of a hypothetical well-appearing, young child presenting with a fever without a source, with relation to his/her anti-pneumococcal vaccination status.

## Methods

### Study population

The sample included representatives from the two different categories of pediatricians operating in Italy (primary care pediatricians, and hospital pediatricians), as well as a group of post-graduate physicians, specializing in pediatrics. All of the pediatricians and pediatric residents registered at the Annual Congress of the Italian Society of Pediatrics held in Pisa, Italy, September 26–29, 2007, were considered eligible for enrollment. This Congress is usually attended by 20–25% of the Italian pediatricians and a similar proportion of pediatric residents [9]. Overall, 1500 questionnaires were distributed.

### Questionnaire and its administration

A self-administered, anonymous questionnaire was distributed to all the physicians at the Congress registration desk. The questionnaire included an explanatory cover letter and was prepared by the authors on the basis of a similar clinical scenario presented to United States pediatricians in a previous study by Wittler and colleagues [10]. The questionnaire was previously pilot-tested on a convenient sample of primary care pediatricians, hospital pediatricians and pediatric residents in order to ensure clarity and ease of administration. The English version of the clinical scenario is shown in Additional File 1. The survey was administered in Italian and translated into English for publication. Responses were anonymous, but demographic (age, gender, graduation year) and professional (primary care pediatrician, hospital pediatrician, or pediatric resident) information was requested.

Participants were asked whether they would send the children home and follow-up ("wait-and see"), would obtain blood tests, or would treat them empirically with antibiotic therapy. Subsequently, pediatricians were asked to mark on a checklist whether they would perform, erythrocyte sedimentation rate, C reactive protein, blood culture, urine analysis or dipstick, urine culture, stool culture, group A β-haemolitic streptococcus rapid test, chest X-ray, abdomen ultrasound scan, or lumbar puncture or whether they would observe and re-evaluate the child later without performing laboratory studies. A blank space was provided in which to write in additional studies thought to be appropriate. A hypothetical blood test exam showing white blood cell count of 17.500/μL (with 45% segmented neutrophils, 15% band forms and 40% lymphocytes) was provided. This scenario was similar to that one previously developed by Wittler and colleagues [10]. Participants were also asked whether, in such scenario, they would send children home and subsequently follow-up, treat them empirically with an antimicrobial or would admit them to hospital. They were also asked to specify which antibiotic they would use for empiric therapy. An additional question was exclusively directed to primary care pediatricians, addressing whether they usually use rapid tests in their offices. A final question explored the participants' general attitudes toward PCV recommendation. The study was in compliance with the Helsinki Declaration and was approved by the Ethical committee of the Meyer Children Hospital, Florence, Italy. All the physicians gave their informed consent to fill out an anonymous survey.

### Statistical methods

Results were stratified by the participants' professional category. Differences in responses regarding the PCV vaccination status evaluated by contingency table analysis with the χ^2 ^or the Fisher's exact test, as appropriate. SPSS^® ^software package ((SPSS 11.5; Chicago, IL) was used, and P < 0.05 was considered as statistically significant.

## Results

### Participants' characteristics

Overall, 690 pediatricians or pediatric residents (46.0% of physicians registered to the Congress) returned the questionnaire. Median age was 48.4 years (interquartile range [IQR]: 35.7–54.0), and 415 (60.06%) were females. Three hundred (46.48%) were primary care pediatricians; 251 (36.38%) were hospital pediatricians and 139 (20.14%) were pediatric residents.

### Initial approach to the well-appearing young child with fever without source, with relation to his/her PCV vaccination status

The proportion of participants who report to "wait-and see" was 60.7% (419/690), if the child was not vaccinated with PCV and increased to 74.2% (n = 512/690) if the child had received PCV (P < 0.0001). Also, physicians would obtain blood tests less frequently in the vaccinated than in the not-vaccinated child (139/690 [20.1%] *vs*. 205/690 [29.7%]). Empiric antibiotic therapy would be started by 3.0% (21/690) of pediatricians/pediatric residents if the child had received PCV *vs*. 7.5% of participants (52/690) if the child was unvaccinated (P < 0.0001) (Table 1). No significant difference was evidenced among physician categories (P = 0.105 considering not-vaccinated child; P = 0.396 considering the vaccinated child, by Pearson χ^2 ^test among categories).

**Table 1:** Change in physicians' self-reported, initial approach to the well-appearing young child, with relation to PCV vaccination status

	***Not vaccinated child***				***Vaccinated child***			
	**Primary care pediatricians**	**Hospital pediatricians**	**Pediatric residents**	**Total**	**Primary care pediatricians**	**Hospital pediatricians**	**Pediatric residents**	**Total**

*Blood examination* *n (%)*	76 (25.3)	82 (32.7)	47 (33.8)	205 (29.7)	55 (18.3)	52 (20.7)	32 (23.0)	139 (20.1)

*Empiric antibiotic treatment* *n (%)*	19 (6.3)	19 (7.6)	14 (10.1)	52 (7.5)	7 (2.3)	7 (2.8)	7 (5.0)	21 (3.0)

*Wait-and see* *n (%)*	197 (65.7)	147 (58.6)	75 (54.0)	419 (60.7)	228 (76.0)	188 (74.9)	96 (69.1)	512 (74.2)

*Not answer* *n (%)*	8 (2.7)	3 (1.2)	3 (2.2)	14 (2.0)	10 (3.3)	4 (1.6)	4 (2.9)	18 (2.6)

P *					0.013	<0.0001	0.021	<0.0001

Note. * vaccinated *vs*. non vaccinated child, by Pearson χ^2 ^test

### Approach to the well-appearing young child with fever without source and white blood cell count = 17,5000/μL, with relation to his/her PCV vaccination status

Participants were asked how they would have managed the child if he/she had shown white blood cell count of 17,500/μL. The proportion of physicians who reported to send the child home with no antibiotic therapy raised from 33.2% (229/690) for the not-vaccinated to 46.5% (321/690) for the vaccinated child (P < 0.0001). A significantly lower proportion of physicians reported to ask for erythrocyte sedimentation rate (P < 0.017), C reactive protein (P < 0.0001), blood culture (P = 0.022), urine analysis/or dipstick (P = 0.028), in the child vaccinated with PCV (Table 2). Investigations would be obtained after 2.2 days of fever (median 2.2; IQR:2.0–3.5 days) with no significant difference regarding the PCV vaccination status. No significant difference was observed among physicians' categories.

**Table 2:** Change in physicians' self-reported approach to the well-appearing young child with white blood cell count of 17,500/μL, with relation to PCV vaccination status

	**Not Vaccinated child** **(n = 690)**	**Vaccinated child** **(n = 690)**	
*Wait-and see (n;%)*	229 (33.2%)	321 (46.5%)	

*Empiric antibiotic therapy (n;%)*	381 (55.2%)	337 (48.8%)	

*Hospital admission (n;%)*	80 (11.6%)	32 (4.7%)	

*P (vaccinated vs. not-vaccinated child)*	<0.0001		

**Investigations (ordered at any time during febrile illness)**	**Not Vaccinated child** **(n = 690)**	**Vaccinated child** **(n = 690)**	**P**

▪ Blood culture	107 (15.5%)	77 (11.2%)	0.022

▪ C reactive protein	468 (67.8%)	394 (57.1%)	<0.0001

▪ Erythrocyte sedimentation rate	163 (23.6%)	126 (18.2%)	0.017

▪ Urine analysis or dipstick	428 (62.0%)	359 (52.0%)	0.028

▪ Urine culture	324 (47.0%)	311 (45.0%)	0.501

▪ Stool culture	12 (1.7%)	14 (2.0%)	0.843

▪ Group A β haemolitic streptococcus rapid test	165 (23.9%)	139 (20.1)	0.104

▪ Chest X ray	56 (8.1%)	41 (5.9%)	0.140

▪ Abdomen ultrasound scan	3 (0.5%)	3 (0.5%)	0.682

▪ Lumbar puncture	7 (1.0%)	6 (0.9%)	1.000

### Preferred antibiotic treatment for empiric therapy

Oral therapy with amoxicillin or amoxicillin/clavulanic acid was the preferred option by 609/690 (88.3%) participants. Ceftriaxone was chosen by 16/690 (2.3%) physicians (Table 3). No significant difference was observed among physicians' categories (P = 0.864).

**Table 3:** Physicians' self-reported preferred antibiotic for therapy empiric treatment.

	**Primary care pediatricians** **n (%)**	**Hospital pediatricians** **n (%)**	**Pediatric residents** **n (%)**	**Total** **N (%)**
*Ceftriaxone i.m./i.v*.	8 (2.7)	7 (2.8)	1 (0.7)	16 (2.3)

*Amoxicillin ± clavulanic acid*	267 (89.0)	218 (86.9)	124 (89.2)	609 (88.3)

*Macrolide*	5 (1.7)	5 (2.0)	2 (1.4)	12 (1.7)

*Per os cephalosporin*	8 (2.7)	10 (4.0)	7 (5.0)	25 (3.6)

*Other*	1 (0.3)	1 (0.4)	0	2 (0.3)

*No answer*	11 (3.7)	10 (4.0)	5 (3.6)	26 (3.8)

Note: i.m.: intramuscular; i.v.: intravenous

### Use of quick tests by primary care pediatricians in the office setting

In general, among the 300 primary care pediatricians who returned the questionnaire, quick test for C reactive protein was reported to be routinely performed by 100/300 (33.33%), urine dipstick test by 293/300 (97.33%), and Group A β haemolitic streptococcus rapid test by 206/300 (68.67%).

### Attitude toward PCV recommendation

Four-hundred-sixty-one (66.81%) participants reported that they to recommend PCV vaccination to all children, while 208 (30.14%) recommend it only in the presence of siblings or day care attendance. Seventeen (2.46%) physicians would never recommend PCV. Finally, 2 (0.29%) pediatricians recommend PCV only in children with a chronic disease, and 2 (0.29%) did not answer. No significant difference was observed among physician categories (P = 0.475).

An additional analysis was performed, considering whether response pattern differs among participants who recommend PCV for all children and those who do not. The wait-and see option has been chosen by 169/229 (73.8%) pediatricians who do not recommend PVC routinely *vs*. 327/461 (70.9%) pediatricians who recommend PCV routinely (p = 0.485) considering the not-vaccinated child, and by 147/221 (66.5%) pediatricians who do not recommend PVC routinely *vs*. 259/461 (56.2%) pediatricians who recommend PCV routinely considering the vaccinated child (p = 0.013).

## Discussion

Results of our survey indicate that pediatricians' self-reported management of the well-appearing young child with a fever without a source considerably change whether (s)he had received PCV or not. The majority (about 60%) of pediatricians/pediatric residents report to "wait-and see" if the child was not vaccinated with PCV, and this proportion significantly increased if the child had received PCV, reaching 76.0%. Parallel to this finding, physicians would choose to obtain blood tests and begin empiric antibiotic therapy less frequently in the vaccinated than in the not-vaccinated child. Empiric antibiotic therapy would be started by about 3.0% of participants if the child had received PCV. The preferred antibiotic treatment for empiric therapy was largely amoxicillin or amoxicillin/clavulanic acid. In the event that the child showed white blood cell count of 17,500/μL (making the risk of bacterial infection higher), one third of participants still would "wait-and see" and send the unvaccinated child home with no therapy. This rate reaches about 45% if the child was vaccinated with PCV, with no significant difference among results obtained by primary care or hospital pediatricians, or pediatric residents. A significantly lower proportion of participants would obtain erythrocyte sedimentation rate, C reactive protein, blood culture, and urine analysis/or dipstick if the child was vaccinated with PCV. Only two thirds of the our participants report to recommend routinely PCV. Additionally, we documented that self-reported use of quick tests (including C reactive protein quick test, group A β-haemolitic streptococcus rapid test, and urine dipstick) is widespread among Italian primary care pediatricians.

In general, our survey results show that management of the well-appearing young child with fever without source is heterogeneous among Italian pediatricians/pediatric residents. Similar data have been previously reported by Wittler and colleagues in 1998 [10], after the proposal of the U.S. guideline for the management of the febrile child [11]. Later, this guideline has been revisited [2]. Subsequently, other algorithms about the management of the febrile child have been published, including some regarding the "wait-and see" approach [12-15]. This approach was the preferred option by our participants, who likely considered the low risk of bacterial infection in this hypothetical child. Nevertheless, the fact that one third of participants still chooses this approach when white blood cell count is 17,500/μL raises concern.

Results similar to ours have been previously reported among United States pediatricians [7,8]. It is worth noting that an unintended effect of PVC widespread on the management of the young children with a fever without a source may include reduced efforts to diagnose urinary tract infection, whose pathogenesis and incidence are not influenced by the child's anti-pneumococcal vaccination status. Possibly, physicians perceive that the PCV vaccinated child is, in general, at minimal risk for all bacterial infections. Indeed, several types of bacterial infections should be ruled out, besides *Streptococcus pneumoniae *infection, including those due to *Neisseria meningitidis, Escherichia coli*, and *Staphylococcus aureus *[14].

The finding that only two-thirds of our participants report to recommend routinely PCV suggests that informative campaign is urgently needed in Italy. This is in strong contrast with results from the United States, where one year after PCV was recommended, nearly all pediatricians had incorporated this vaccine [15,16].

The preferred antibiotic treatment for empiric therapy was oral amoxicillin. The low use of macrolides is justified in Italy, since bacterial resistance to this class of drugs is wide-spread, as well as in other European countries [17]. The choice of an oral antibiotic with respect to ceftriaxone is in contrast with current guidelines, recommending ceftriaxone or another third generation cephalosporin [14]. Pediatricians might have considered the results of studies showing equal efficacy of oral and parenteral antibiotics in preventing severe bacterial infections in well-appearing children with *Streptococcus pneumoniae *occult bacteraemia [18]. Possibly, they considered the reduced discomfort from an oral course of antibiotics. However, it must be remembered that, according to the current guidelines, in a child older than three months, presenting with a fever and suspected serious bacterial infection, antibiotics should cover *Neisseria meningitidis, Streptococcus pneumoniae, Escherichia coli, Staphylococcus aureus *and *Haemophilus influenzae *type b. Thus, ceftriaxone or cefotaxime should be considered as the first line therapy [14].

Our investigation has potential limitations. First, our results may not generalize to all pediatricians nationwide. Participans included in the study constituted approximately 10% of all the about 7500 Italian pediatricians working in Italy in 2007 (Italian Pediatric Society secretariat: personal communication), and were all attending the Annual Congress of the Italian Pediatric Society. Therefore, our study population may be not representative of all Italian pediatricians/pediatric residents. Second, it is well known that self-reported behavior can be misleading since some participants might not complete the survey as carefully as they would provide medical care [19]. The fact that other two similar surveys conducted in the United States [7,8] and one observational study on children attending an emergency department in Spain [20] documented results similar to ours further corroborate our findings. Finally, the attitude toward urine testing has been reported to vary among pediatricians, and a potential limitation of our study is that results may have differed if the child's age was younger or if the gender was specified [21].

Encouraging results of large epidemiological studies indicate substantial reduced risk of invasive pneumococcal infection in children vaccinated with PCV [22]. The effects of decreased rates of pneumococcal disease on routine clinical practice are potentially significant [23]. However, pneumococcal serotype replacement has been documented [24], and, to date, evidence is lacking to modify algorithms for the management of the febrile child, considering his/her PCV vaccination status.

## Conclusion

Italian pediatricians may obtain less blood and urine tests and prescribe less empiric antibiotic treatments in well-appearing young febrile children if they have received PCV. However, until more data are available regarding the actual risk of bacterial infection in children vaccinated with PVC in developed countries, this approach should not be sanctioned.

## Competing interests

The authors declare that they have no competing interests.

## Authors' contributions

EC, LG, FB and EV initiated the study, the design, and the data collection. EC, EV and MdM performed the statistical analysis and were involved in the interpretation of the results. EC, LG, NP and Mdm wrote the manuscript. All authors read and approved the final manuscript.

## Pre-publication history

The pre-publication history for this paper can be accessed here:



## Supplementary Material

Additional file 1 **Clinical scenario and questionnaire.** Click here for file
