# Supplementary material for: Self-reported pediatricians' management of the well-appearing young child with fever without a source: first survey in an European country in the anti-pneumococcal vaccine era
Source: BMC Public Health. 2009 Aug 19;9:300. doi: 10.1186/1471-2458-9-300 (PMC2736943; doi:10.1186/1471-2458-9-300)
Supplement: Additional file 1 — Clinical scenario and questionnaire. [file 1471-2458-9-300-S1.doc]

**Appendix 1. Clinical scenario and questionnaire**

*Two previously healthy children, aged 12 months, are brought to your office/clinic with the complaints of fever first noted the previous evening. They have no cough, nasal congestion, emesis or diarrhea. They do not attend day care, their vaccinations have been appropriately maintained and they parents and older siblings are well. Their past medical and family histories are unremarkable and they have no allergies. Their rectal temperatures on arrival were 40.3°C and, after acetaminophen treatment, temperatures are now 39.2 °C. Other vital signs are normal and their heights, weights, and head circumferences are on track. They lie listlessly in their mothers’ laps but respond appropriately for their ages. Although somewhat irritable when examined, they are easily consoled and breast-fed avidly afterward. They appear well-hydrated, they have no signs of meningeal inflammation or respiratory distress and your careful examination reveals no evidence of focal infection in any of them.* ***Patient 1*** *has been vaccinated with heptavalent pneumococcal conjugate vaccine (PCV) (three doses).* ***Patient 2*** *has not been vaccinated with* *PCV*

1. ***How would you manage the two described children? (check only one answer )***

***Not- Immunized child Immunized - child***

*a) I would send him/her home and follow-up a) I would send him/her home and follow-up*

*b) I would obtain blood examination immediately b) I would obtain blood examination*

*immediately*

*c) I would start empiric antibiotic treatment c) I would start empiric antibiotic*

*treatment*

2) **How you would manage these children if blood tests would show white blood cell count of 17.500/μL with 45% segmented neutrophils, 15% band forms and 40% lymphocytes?**

***Not- Immunized child Immunized - child***

*a) I would send him/her home and follow-up a) I would send him/her home and follow-up*

*b) I would send him/her home with b) I would send him/her home with*

*an empiric antibiotic therapy an empiric antibiotic therapy*

*c) I would admitted him/her to hospital c) I would admitted him/her to hospital*

***Not- Immunized child Immunized - child***

*In this case, I would obtain investigations after In this case, I would obtain investigations after*

*________days from fever onset, obtaining: ________days from fever onset, obtaining:*

*(check one or more answers) (check one or more answers)*

- erythrocyte sedimentation rate, - erythrocyte sedimentation rate,

- C reactive protein, - C reactive protein,

- blood culture, - blood culture,

- urine analysis or dipstick, - urine analysis or dipstick,

- urine culture, - urine culture,

- stool culture, - stool culture,

- Group A β haemolitic streptococcus - Group A β haemolitic streptococcus

rapid test, rapid test,

*-* chest X-ray, *-* chest X-ray,

- abdomen ultrasound scan, - abdomen ultrasound scan,

- lumbar puncture - lumbar puncture

-other __________________ -other __________________

**3)** **Which kind of antibiotic would you choose for empiric treatment ?**

1. *ceftriaxone*
2. *amoxicillin or amoxicillin/clavulanic acid*
3. *macrolide*
4. *per os cephalosporin*
5. *other_________________*

**4)** **Which kind of rapid tests do you usually use in your office ? (exclusively for primary care pediatricians)**

1. *quick test for C reactive protein*
2. *urine dipstick*
3. *Group A β haemolitic streptococcus* *rapid test*
4. other __________________________________

**5)**  **Do you recommend PCV to children**

1. *always*
2. *never*
3. *only in cases with siblings or attending day care*
4. *other _____________________________________________*
